# Wdr68 Requires Nuclear Access for Craniofacial Development

**DOI:** 10.1371/journal.pone.0054363

**Published:** 2013-01-22

**Authors:** Bingyan Wang, Diana Doan, Yanett Roman Petersen, Estibaliz Alvarado, Gregory Alvarado, Ajay Bhandari, Aditya Mohanty, Sudipta Mohanty, Robert M. Nissen

**Affiliations:** Department of Biological Sciences, California State University Los Angeles, Los Angeles, California, United States of America; Montana State University, United States of America

## Abstract

Wdr68 is a highly conserved scaffolding protein required for craniofacial development and left-right asymmetry. A Ras-Map3k-Wdr68-Dyrk1 signaling relay may mediate these and other diverse signaling events important in development and disease. While the sub-cellular localization of Wdr68 has been shown to be dependent on that of its interaction partners, it is not clear where Wdr68 activity is required during development. Here we show that while a GFP-Wdr68 fusion functionally substituted for craniofacial development in the zebrafish, that a Nuclear Export Signal (NES) fusion protein (GFPNESWdr68) failed to support craniofacial development. As control for NES activity, we show that while GFP-Wdr68 exhibited a pan-cellular distribution in C2C12 cells, the GFPNESWdr68 fusion predominantly localized to the cell cytoplasm, as expected. Interestingly, while GFP-Wdr68 and RFP-Dyrk1a co-localized to the cell nucleus as expected based on the known sub-cellular localization for Dyrk1a, we found that the GFPNESWdr68 fusion redistributed RFP-Dyrk1a to the cell cytoplasm potentially disconnecting the Ras/Dyrk1 signal relay from further downstream targets. Consistent with a nuclear role in gene regulation, we also found that while a transcriptional activation domain fusion, CebpFlagWdr68, functionally substituted for endogenous Wdr68 for craniofacial development, that a transcriptional repression domain fusion, MadFlagWdr68, failed to support craniofacial development. Dyrk1b is required for *myogenin (myog)* expression in differentiating mouse C2C12 cells and here we report that *wdr68* is also important for *myog* expression in differentiating C2C12 cells. Using a C2C12 cell *myog* promoter-reporter system, we found that Wdr68 overexpression increased reporter activity while moderate expression levels of MadFlagWdr68 interfered with reporter activity. Taken together, these findings support a nuclear role for Wdr68-containing complexes.

## Introduction

Wdr68/Dcaf7 (hereafter referred to as Wdr68) is a WD40 repeat domain-containing protein that is highly conserved from animals to plants and is involved in diverse cellular processes. In the zebrafish *Danio rerio*, Wdr68 is required for craniofacial development upstream of *endothelin-1 (edn1)* expression [1], and left-right asymmetry upstream of *south paw (spaw)* expression [2]. In *Caenorhabditis elegans*, the Wdr68 paralogs Swan-1 and Swan-2 are important for the osmotic stress response [3]. The Petunia ortholog AN11 is important for regulation of anthocyanin biosynthesis [4]. Interestingly, the Arabidopsis ortholog LWD1, which is 58% identical to Wdr68, is important for expression of clock genes and localizes to their promoters in vivo [5]. While parallels between such diverse processes are unclear, the ability of the drosophila ortholog CG14614 to substitute for *wdr68* in zebrafish craniofacial development suggests the molecular function of *wdr68* is highly conserved [1].

WD40 repeat domains are not known to possess catalytic activity but instead serve as protein interaction modules [6]. Wdr68 can physically interact with several kinases including two closely related members of the Dual-specificity tyrosine-regulated kinase gene family, Dyrk1a and Dyrk1b [7]. The *dyrk1b* gene was originally identified as over-expressed in colon cancers [8]. Dyrk1b is now recognized for its role as a downstream effector of oncogenic Ras that promotes pancreatic cancer cell survival [2], [9], [10], [11]. Dyrk1b is detected in both the nucleus and cytoplasm suggesting the existence of a Wdr68-Dyrk1b protein complex capable of shuttling between the cytoplasm and nuclear compartment [7], [12]. Wdr68 also binds MEKK1 thereby serving to physically bridge signaling from activated MEKK1 to downstream kinases such as Dyrk1a and Dyrk1b [3], [13]. Since MEKK1 interacts with the upstream activator Ras [14], Wdr68 appears to serve as a scaffolding protein in a Ras-MEKK1-Wdr68-Dyrk1 signaling cascade by physically linking upstream and downstream kinases such as MEKK1 and Dyrk1a/b, respectively. Consistent with this Ras-MAPK variant signaling pathway model, a Ras-Dyrk1b pathway is known to downregulate autocrine Hedgehog signaling in pancreatic ductal adenocarcinomas [11].

Studies in mouse C2C12 cells revealed that Dyrk1b acts as a differentiation switch during muscle development [15], [16]. Endogenous Dyrk1b is up-regulated under the mitogen deprivation conditions that induce differentiation via transcriptional mechanisms regulated by the Rho family of small GTPases, RhoA and Cdc42 [15], [17], [18]. Dyrk1b is important for the expression of a variety of muscle-specific functional proteins such as troponin T and muscle myosin heavy chain (MHC) [15]. The depletion of *dyrk1b* by small interfering RNA blocks *myogenin (myog)* expression [15]. Dyrk1b phosphorylates class II histone deacetylases (HDACs) at a conserved serine site within their nuclear localization signal, reducing their nuclear accumulation [12], [19]. The myocyte enhancer factor-2 (MEF2) transcription factors are bound by the repressor HDAC5 [12]. When no longer concentrated in the nucleus, phosphorylated HDAC5 is unable to inhibit MEF2s [19]. The release of MEF2s allows transcription of *myog*, which subsequently regulates muscle differentiation [19]. Thus, through inhibiting a MEF2 inhibitor, *dyrk1b* functions as a co-activator for *myog* expression [19]. Dyrk1b can also physically interact with other transcription factors such as HNF1a, suggesting the existence of more general nuclear Dyrk1b-containing transcriptional co-regulatory complexes [20].

Taken together, these previous reports suggest the existence of an evolutionarily conserved variant Ras-Dyrk1 transcriptional co-regulatory cascade that is important in diverse organisms for multiple context-dependent signaling outcomes. However, the majority of these findings derive from studies on isolated cell lines analyzed in vitro. Therefore, we tested certain predictions of this model using the zebrafish as a complement to analyses in the C2C12 cell line. First, we found that a constitutively cytoplasmic form of Wdr68 is incapable of substituting in the zebrafish for craniofacial development. Second, the fusion of a transcriptional repression domain to Wdr68 impaired function while the fusion of a transcriptional activation domain to Wdr68 was permissive to craniofacial development. We found evidence of similar behavior in vitro. Third, we found that *wdr68* is important for *myog* expression in the C2C12 cell model. These findings are consistent with the existence of a nuclear Wdr68-Dyrk1 transcriptional co-activating complex.

## Results

**Figure 1:**
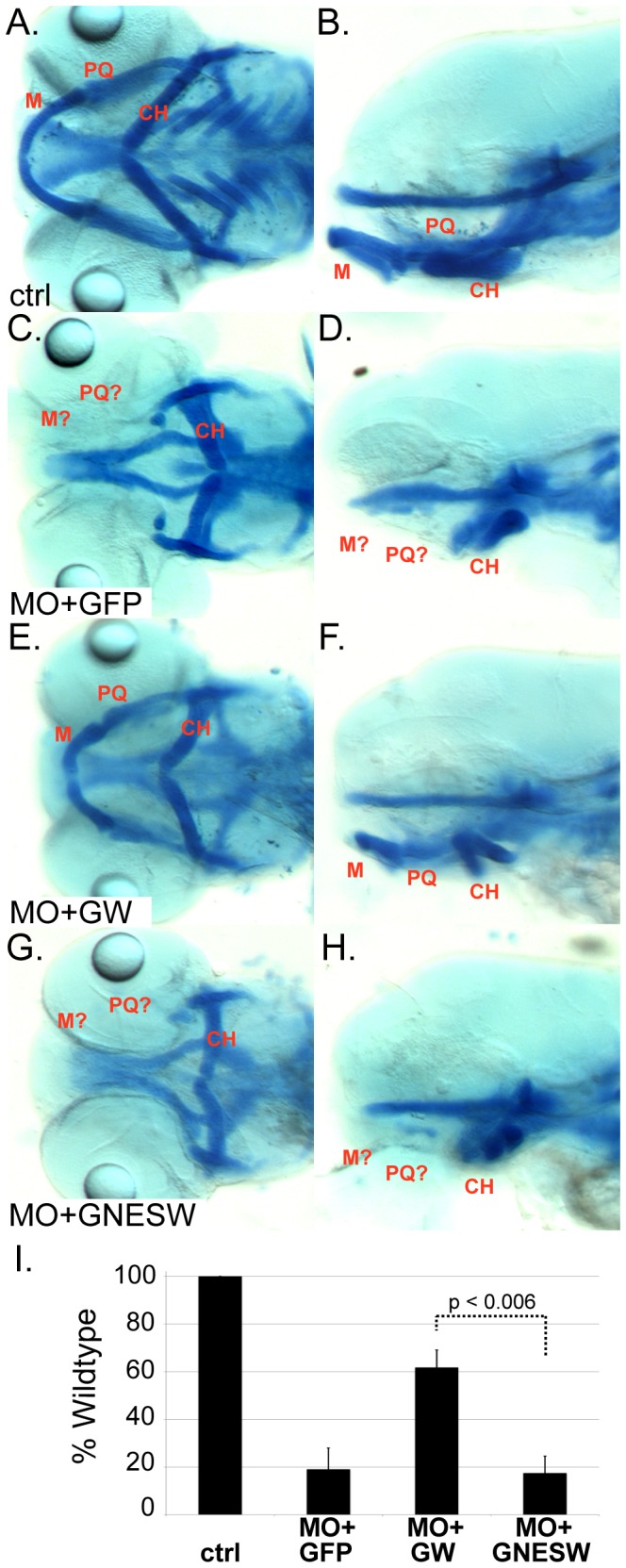
Nuclear access is required for Wdr68 function in craniofacial development. A–H) Alcian blue stained animals. A, C, E, G) Ventral view with the Meckel’s (M), palatoquadrate (PQ) and ceratohyal (CH) labeled in red. B, D, F, H) Lateral view. A, B) Wildtype control-MO (ctrl) injected animals. C, D) Severe craniofacial phenotype of *wdr68*-MO injected animals co-injected with GFP mRNA (MO+GFP) that fails to rescue the craniofacial defects. E, F) Rescue of the craniofacial defects of *wdr68*-MO animals co-injected with GFP-Wdr68 mRNA (MO+GW). G, H) Severe craniofacial defects of *wdr68*-MO animals co-injected with GFPNESWdr68 mRNA (MO+GNESW) that fails to rescue the craniofacial defects. I) Graph summarizing the results of three injection trials. In aggregate, n = 50/50 (100%) for control animals, n = 47/257 (18%) for MO+GFP animals, n = 212/350 (61%) for MO+GW animals, and n = 71/385 (18%) for MO+GNESW animals.

In zebrafish, the Meckel’s (M) cartilage serves as the embryonic lower jaw and the palatoquadrate (PQ), in particular the ptergoid process (PTP) of the PQ, serves as the embryonic upper jaw [21], [22]. We previously identified the *wdr68* gene as essential for craniofacial development partly due to its essential role for endothelin-1 (edn1) expression [1]. To examine more closely a possible role in transcriptional regulation, we tested whether a constitutively cytoplasmic Wdr68 protein can substitute for the wildtype gene during development. To test this, we embedded the Nuclear Export Sequence (NES) of MEK2 between GFP and Wdr68 to yield a GFPNESWdr68 fusion. The 13 amino acid NES of MEK2 is particularly efficient in export assays and identical between fish and human [23]. A GFPWdr68 fusion was included as positive control for rescue. RNA rescue assays were performed by injecting the various test RNAs into wildtype embryos co-injected with antisense *wdr68* morpholino (*wdr68*-MO) to knockdown endogenous *wdr68* (Figure 1, Table S1). The *wdr68*-MO has been previously characterized and yields less phenotypic variability than do *wdr68^hi3812^/wdr68^hi3812^* animals [1]. Animals injected with a control morpholino appeared indistinguishable from uninjected wildtype animals in which the M and PQ are readily identifiable (Figure 1A, B). Only 18% of animals co-injected with *wdr68*-MO and negative control GFP mRNA displayed normal M and PQ cartilages indicating a failure of RNA rescue (Figure 1C, D). In contrast, 61% of animals injected with *wdr68*-MO and the positive control GFPWdr68 (GW) mRNA displayed normal M and PQ cartilages indicating a substantial degree of phenotypic rescue (Figure 1E, F). However, only 18% of animals injected with GFPNESWdr68 (GNESW) mRNA displayed normal M and PQ cartilages indicating a failure of RNA rescue (Figure 1G, H), similar to that observed for the negative control (compare to Figure 1C, D). Figure 1I summarizes the RNA rescue results from three independent trails in graph form.

**Figure 2:**
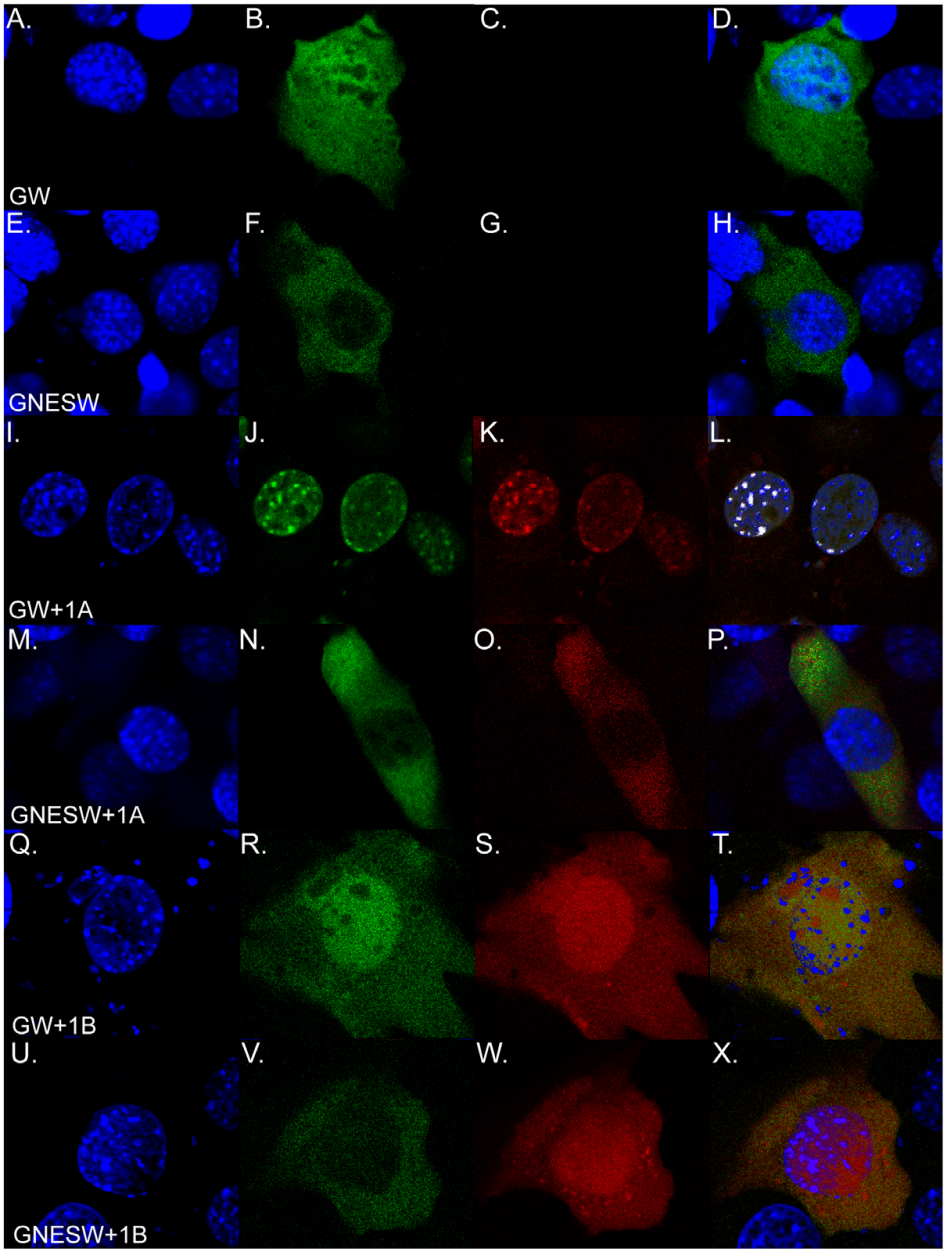
The GFPNESWdr68 fusion redistributes interaction partner Dyrk1a from the nucleus to the cytoplasm in C2C12 cells. A, E, I, M, Q, U) DAPI stained cell nuclei. B, F, J, N, R, V) GFP fusion proteins. C, G, K, O, S, W) mRFP1 fusion proteins. D, H, L, P, T, X) Overlays of blue, green and red channels. A–D) pan-cellular distribution of GFPWdr68 (GW) alone. E–H) Cytoplasmic restriction of GFPNESWdr68 (GNESW) alone. I–L) Redistribution of GW to the cell nucleus when co-expressed with mRFP1-Dyrk1a. M–P) Redistribution of mRFP1-Dyrk1a when co-expressed with GNESW. Q–T) Pan-cellular distribution of GW when co-expressed with pan-cellular mRFP1-Dyrk1b. U–X) Cytoplasmic restriction of GNESW when co-expressed with pan-cellular mRFP1-Dyrk1b.

To verify function of the NES fusion, we transfected mouse C2C12 cells with GFPWdr68 or GFPNESWdr68 alone and in combination with RFP-Dyrk1a or RFP-Dyrk1b (Figure 2). The GFPWdr68 fusion protein displayed a pan-cellular distribution in C2C12 cells that was not readily distinguishable from that of GFP alone (Figure 2A–D, and data not shown). In contrast, the GFPNESWdr68 fusion protein was predominantly excluded from the cell nucleus in C2C12 cells (Figure 2E–H). Consistent with previous reports [1], [3], the GFPWdr68 fusion protein was predominantly localized to the cell nucleus when co-expressed with the RFP-Dyrk1a fusion protein (compare Figure 2I–L to Figure 2D). Notably, the RFP-Dyrk1a fusion protein redistributed to the cell cytoplasm when co-expressed with the GFPNESWdr68 fusion protein (compare Figure 2M–P to Figure 2L). We also examined whether the Wdr68 fusions exerted any influence over the localization of a RFP-Dyrk1b fusion protein that is also known to physically interact with Wdr68 [2], [3], [7]. In the absence of Wdr68, the RFP-Dyrk1b fusion protein displayed a pan-cellular distribution in C2C12 cells similar to that observed when co-expressed with GFPWdr68 (Figure 2Q–T, and data not shown). Co-expression of GFPNESWdr68 with RFP-Dyrk1b revealed a similar nuclear exclusion for GFPNESWdr68 without perturbing nuclear RFP-Dyrk1b (Figure 2U–X). We also examined the subcellular localization of GFPWdr68 and GFPNESWdr68 in zebrafish embryos and observed a similar pattern of nuclear occupancy for GFPWdr68 and exclusion for GFPNESWdr68 (Figure S1). We speculate that the inability of the GFPNESWdr68 fusion to alter the subcellular localization of Dyrk1b might arise from stronger or more dynamic signals controlling the subcellular localization of Dyrk1b relative to Dyrk1a. Nonetheless, the displacement of GFPNESWdr68 from the nucleus would, in principle, disrupt any potential MEKK1-Wdr68-Dyrk1a/b nuclear signaling complexes.

**Figure 3:**
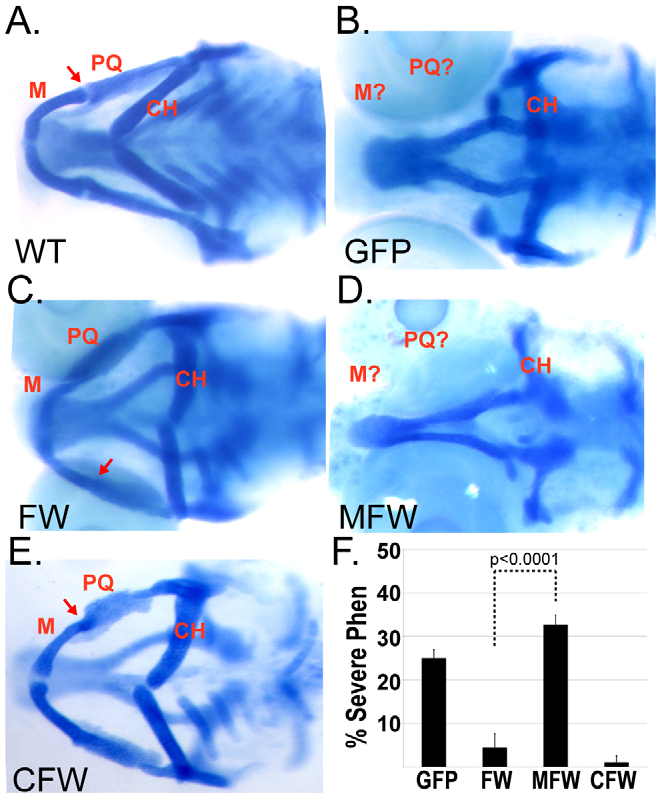
Fusion of Wdr68 to the Mad repression domain is not tolerated in vivo. A–E) Ventral views of alcian blue stained animals with the Meckel’s (M), palatoquadrate (PQ) and ceratohyal (CH) labeled in red. A) Wildtype sibling. Red arrow indicates the M-PQ jaw joint. B) Severe craniofacial defects in *wdr68* ^hi3812^ animals and failure of GFP mRNA to rescue. C) Rescue of the severe craniofacial defects by injection of FlagWdr68 (FW) mRNA. Red arrow indicates the mild M-PQ joint fusion phenotype. D) Severe craniofacial defects in *wdr68* ^hi3812^ animals and failure of MadFlagWdr68 (MFW) mRNA to rescue. 52/52 mutants genotyped were *wdr68* ^hi3812^ homozygotes. E) Rescue of the severe craniofacial defects by injection of CebpFlagWdr68 (CFW) mRNA. Red arrow indicates the mild M-PQ joint fusion phenotype. F) Graph summarizing the results of four injection trials. In aggregate, n = 112/452 (25%) for GFP animals, n = 16/352 (4.5%) for FW animals, n = 96/296 (32%) for MFW animals, and n = 2/149 (1.3%) for CFW animals.

The Wdr68 interaction partner Dyrk1b functions like a co-activator for *myogenin (myog)* expression by inhibiting HDAC5 and can physically interact with transcription factors, such as HNF1a, suggesting potentially direct roles in the regulation of transcription [15], [20]. As a putative member of at least one transcriptional co-regulatory complex, we sought to determine whether transcriptional repression or activation domain fusions to Wdr68 would impair craniofacial development. A fusion of the Flag epitope tag to the N-terminus of Wdr68, FlagWdr68 (FW), can functionally substitute for endogenous Wdr68 for zebrafish craniofacial development [1]. The N-terminal 26 kD GFP fusion to Wdr68 also suggests that modification of the N-terminus does not generally interfere with Wdr68 function (Figure 1E,F). To further probe a potential role in transcriptional regulation, we created a fusion between the FlagWdr68 construct and the 35-residue transcriptional repression domain from the Mad1 protein that recruits HDAC1/2 activity [24], [25]. If Wdr68 functions in nuclear complexes involved in the activation of various target genes important for craniofacial development, then the MadFlagWdr68 (MFW) fusion should fail in the RNA rescue assay. The zebrafish Cebp1 transcriptional activator has an 87-residue activation domain that functions when transferred to GAL4 [26], [27]. As a positive control, we generated a CebpFlagWdr68 (CFW) fusion that, if anything, might be expected to further enhance transcriptional activation and thus not interfere with craniofacial development. In order to detect any potential dominant negative effects of the MFW construct, RNA rescue assays were performed by injecting the various test RNAs into embryos from crosses of heterozygous *wdr68^hi3812^*/+ carriers (Figure 3, Table S2). According to Mendelian inheritance, one would expect approximately 75% wildtype embryos and 25% phenotypic embryos from these crosses. Significant reduction of the percent of phenotypic animals below 25% by a test mRNA indicates phenotypic rescue. A representative wildtype sibling control is shown for reference (Figure 3A). Reflecting normal Mendelian ratios, 25% of GFP-injected negative control animals from crosses of heterozygous *wdr68^hi3812^*/+ carriers displayed the severe craniofacial phenotype (Figure 3B). Consistent with previous findings [1], we found that only 4.5% of FlagWdr68-injected animals from crosses of heterozygous *wdr68^hi3812^*/+ carriers displayed severe craniofacial defects indicating successful RNA rescue (Figure 3C). In contrast, MadFlagWdr68-injected animals from crosses of heterozygous *wdr68^hi3812^*/+ carriers failed to rescue craniofacial development (Figure 3D). To control for the possibility that fusions to the N-terminus of Wdr68 might generally interfere with protein function, we also tested the 87-residue Cebp1 fusion construct. Only 1.3% of CebpFlagWdr68-injected animals from crosses of heterozygous *wdr68^hi3812^*/+ carriers displayed severe craniofacial defects indicating successful RNA rescue (Figure 3E). It should be noted that phenotypic rescue is incomplete. Rescued animals displayed a relatively mild M-to-PQ joint fusion defect but were still readily identified as distinct from wildtype animals (Figure 3C, E compare the area denoted by the red arrow to 3A), as previously described [1]. Figure 3F summarizes the results of three independent trials in graph format.

The only known biochemical functions of Wdr68 are physical interaction with partner proteins such as Dyrk1a and Dyrk1b [2], [3], [7]. Although our findings argue against the possibility that N-terminal modifications of Wdr68 generally impair Wdr68 function, we sought to independently assess the biochemical function of the MadFlagWdr68 construct. Using Dyrk1b and FlagWdr68 as the positive control, co-immunoprecipitation (Co-IP) experiments were performed using an anti-Flag antibody and in vitro translated proteins to determine whether the MadFlagWdr68 fusion protein was still capable of interaction with Dyrk1b (Figure S2). The 35-S-methionine labeled protein inputs are shown (Figure S2, lanes 1–4). As expected we were unable to detect an interaction between the negative control Luciferase protein and FlagWdr68 (Figure S2, lane 5). In contrast, we readily detected the interaction between Dyrk1b and FlagWdr68 (Figure S2, lane 6). As an additional negative control testing for unspecific binding of Dyrk1b to the Co-IP beads, we were unable to detect Dyrk1b in the absence of Wdr68 (Figure S2, lane 7). We readily detected an interaction between Dyrk1b and the MadFlagWdr68 fusion protein (Figure S2, lane 8), and were unable to detect an interaction between negative control Luciferase and MadFlagWdr68 (Figure S2, lane 9). These findings indicate that MadFlagWdr68 is a stable protein in vitro and retains the ability to specifically and physically interact with Dyrk1b. Taken together with the Figure 3 results, these findings are consistent with a model in which Wdr68 may act as a member of a transcriptional co-regulatory complex important for craniofacial development.

**Figure 4:**
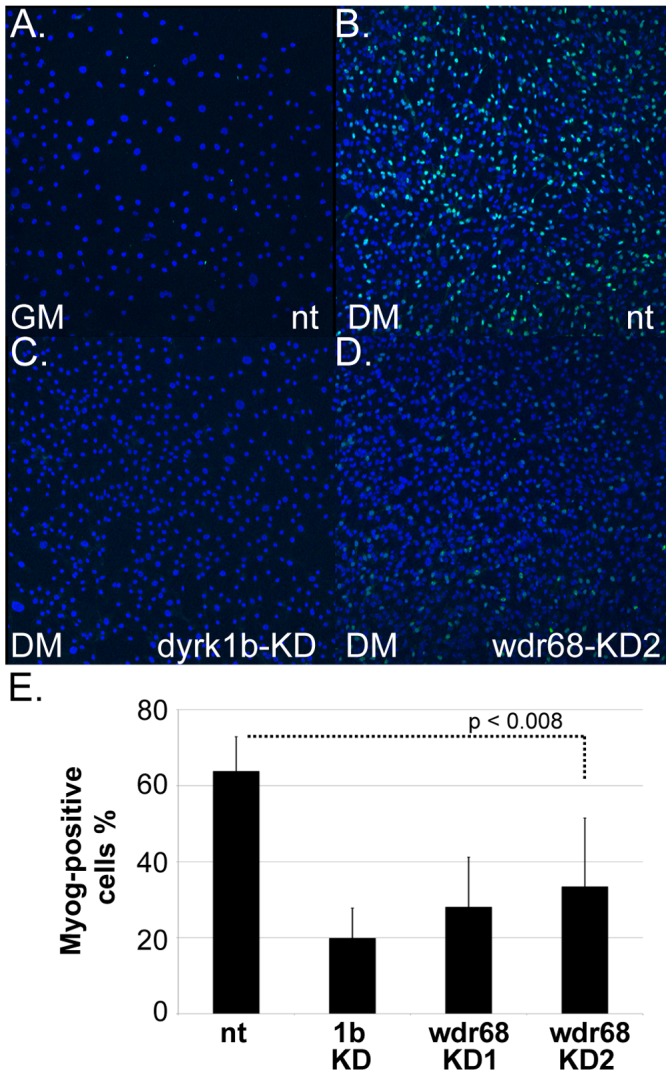
Wdr68 is important for Myog expression in differentiating C2C12 cells. A–D) Overlay of DAPI and Alexa-488 detection of Myog expression. A) Non-target (nt) control cells in growth medium (GM). B) nt control cells expressing Myog after 48 hours in differentiation medium (DM). C) Severely reduced expression of Myog in *dyrk1b*-KD cells. D) Reduced expression of Myog in *wdr68*-KD2 cells. E) Graph summarizing the number of Myog+ cells results for eight independent trials.

In mammals, the DNA-binding transcriptional regulator *myog* is expressed predominantly in skeletal muscle cells and functions during differentiation in the transition from myoblast to myotube [28], [29]. The mouse C2C12 myoblast cell line model of muscle differentiation is a well-characterized and homogenous in vitro system for the analysis of promoter-reporter assays. Since Dyrk1b is required for *myog* expression and myotube differentiation in C2C12 cells [15], we sought to expand our studies of *wdr68* activity into C2C12 cells as a homogenous system for biochemical analyses. Using RT-PCR, we verified that *wdr68* is expressed in C2C12 cells (Figure S3). Dyrk1b is required for Myog expression and *dyrk1b*-knockdown (*dyrk1b*-KD) C2C12 cells have been previously described [12], [15]. Using a lentiviral-based method for shRNA delivery and quantitative western blotting, we also generated *wdr68*-knockdowns (*wdr68*-KD1 and *wdr68*-KD2) that yielded 50% to 78% reductions in Wdr68 protein levels relative to non-target (nt) control cells (Figure S4). We then examined the induction of Myog in differentiating nt control, *dyrk1b*-KD, *wdr68*-KD1 and *wdr68*-KD2 C2C12 cells (Figure 4A–D). The induction of Myog expression was readily apparent after 48 h in differentiation medium (compare Figure 4A to 4B). The nt control cells were 64% +/−9% positive for Myog expression by immunocytochemistry (Figure 4B and 4E). In contrast, the *dyrk1b*-knockdown (1b-KD) cells were only 20% +/−8% positive for Myog expression (Figure 4C and 4E). Likewise, two different shRNA constructs against *wdr68* were tested (Figure 4D and 4E). The *wdr68*-KD1 cells were 28% +/−13% positive and the *wdr68*-KD2 cells were 33% +/−18% positive for Myog expression (Figure 4E). These findings indicate that the C2C12 cell line is a valid system for studying *wdr68* activity and that *wdr68* is important for robust *myog* expression.

**Figure 5:**
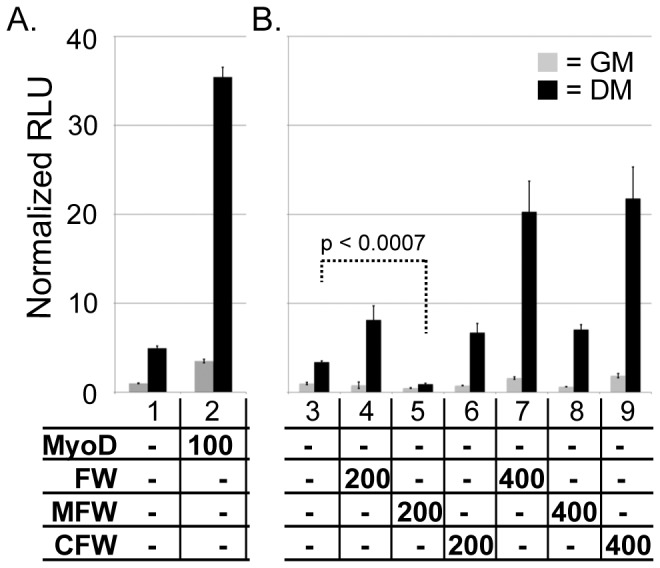
Wdr68 and derivatives modulate transcriptional activation of a *myog* promoter-reporter construct in C2C12 cells. C2C12 cells transfected with myog-luciferase and SV40-renilla reporters. Grey columns are cells in growth medium (GM). Black columns are cells in differentiation medium (DM). The y-axis units are the same for parts A and B. A) Column pairs 1) empty pCS2+ expression vector control showing endogenous basal levels of reporter induction, 2) 100 ng MyoD further enhances reporter activity 7-fold above control. B) Column pairs 3) basal control, 4) 200 ng FlagWdr68 (FW) further enhances reporter activity 2.4-fold above control, 5) 200 ng MadFlagWdr68 (MFW) represses reporter activity to 27% of control, 6) 200 ng CebpFlagWdr68 (CFW) further enhances reporter activity 2-fold, 7) 400 ng FW further enhances reporter activity 6-fold, 8) 400 ng MFW enhances reporter activity only 2-fold, 9) 400 ng CFW further enhances reporter activity 6.4-fold.

MyoD and Myf5, together with MEF2 family proteins and other general muscle-specific factors, regulate skeletal muscle commitment and induce *myog* expression [28], [29]. Dyrk1b enhances MEF2C induction of *myog* promoter-reporter constructs in C2C12 cells [12]. In light of our findings with *wdr68* knockdown cells, we examined whether the MadFlagWdr68 and CebpFlagWdr68 constructs might influence *myog* promoter-reporter activity in transiently transected C2C12 cells. As a control for reporter induction, we tested MyoD and found that it enhanced reporter activity an additional 7-fold in differentiating C2C12 cells (Figure 5A, compare column pair 2 DM to column pair 1 DM). We found that FlagWdr68 and CebpFlagWdr68 enhanced reporter activity an additional 2-fold in differentiating C2C12 cells (Figure 5B, compare column pair 4 DM and column pair 6 DM to column pair 3 DM). In contrast, we found that moderate levels of expression of the MadFlagWdr68 construct repressed reporter activity to 27% of basal (Figure 5B, compare column pair 5 DM to column pair 3 DM). At higher levels of expression, the MadFlagWdr68 construct also performed less well than either the FlagWdr68 or CebpFlagWdr68 constructs (Figure 5, column pairs 7–9). The discrepancies between the amounts of transfected plasmids required to observe regulatory effects may be a reflection of the fact that the pCS2+ vector, used for expression of the various Wdr68 derivatives, and the pEMSV vector, used for expression of MyoD, contain different promoters (Figure 5A, B). Overall, these findings are consistent with those found in vivo (Figure 3).

## Discussion

The sub-cellular localization of Wdr68 depends on that of its interaction partner [3], [30]. In the presence of Dyrk1a, Wdr68 is predominantly localized to the cell nucleus in C2C12 cells (Figure 2). In contrast, co-expression with Dyrk1b yields a pan-cellular distribution of Wdr68. Although the intersection of these sub-cellular distributions suggests a common role within the nucleus, our findings in the zebrafish demonstrate that nuclear access is indeed a requirement for Wdr68 function in vivo (Figure 1, S1).

Dyrk1b functions as a co-activator for *myog* expression by blocking HDAC5 association with MEF2 transcription factors [12], [15], [16] [31]. Wdr68 is required for *edn1* expression [1]. Interestingly, *mef2ca* is an important mediator of Edn1 pathway signaling [32]. Our findings in C2C12 cells indicate *wdr68* is also important for expression of *myog.* Together these findings suggest *wdr68* may function both upstream of *edn1* expression as well as to facilitate events downstream of *edn1* expression.

Chromatin immunoprecipitation studies on the Wdr68 ortholog LWD1, which is 58% identical to Wdr68, revealed association between LWD1 and the promoters of several LWD1-dependent genes [5]. Given our findings that Wdr68 is required in the nucleus in vivo (Figure 1, S1), can enhance a *myog* promoter-reporter in vitro, and MFW can repress *myog* reporter activity in vitro (Figure 5, 200 ng conditions), it is attractive to speculate a similar promoter-proximal role in gene regulation for Wdr68-containing complexes. However, the repressive effects of the MFW construct were lost at higher expression levels suggesting the fusion may instead interfere with Wdr68 function or stability in an unspecific manner (Figure 5, 400 ng conditions). Likewise, efforts to detect even partial dominant negative activity from the MFW construct in vivo were unsuccessful (Figure 3D and data not shown). The dependence of Wdr68 sub-cellular localization on other factors also makes the general applicability of a promoter-proximal role in gene regulation for Wdr68-containing complexes unclear. Future biochemical studies on Wdr68-containing complexes in the homogenous C2C12 cell system would shed additional light on these possibilities.

Cells with reduced levels of wdr68 expression have been reported to display impaired growth rates [30]. Although we noted an initial growth delay in our C2C12 wdr68-KD cells, differences between control and knockdown cells were not statistically significant (data not shown). It is possible that our method for achieving stable knockdowns may have selected against cells displaying more pronounced growth defects.

To date, Wdr68 has been reported to physically interact with only a single member of the MAP3K family, MEKK1 [3], and MEKK1 mutants are born live without jaw defects [33]. Therefore, it is unlikely that MEKK1 represents the major upstream signaling partner for Wdr68 in craniofacial development. Notably, there are over 21 different members of the MAP3K family. Our findings that wdr68 is important for *myog* expression in the C2C12 cell model (Figure S3, S3, 4) enables the future exploitation of this homogeneous cell culture system for biochemical characterization of Wdr68-containing complexes to potentially identify additional MAP3K-like interaction partners. Future studies will be needed to identify and systematically test additional upstream partners for potential roles in craniofacial development.

## Methods

### Ethics Statement

This study was carried out in strict accordance with the recommendations in the Guidelines for Use of Zebrafish in the NIH Intramural Research Program. The protocol was approved by the Institutional Animal Care and Use Committee at California State University Los Angeles (permit 11-3).

### Animal Husbandry

Wildtype TAB14 and *wdr68* ^hi3812^ carrier strains were maintained essentially as described [1].

### Plasmid Construction

Plasmids containing the various fusions were constructed using standard molecular biology techniques. The pCS2+FlagWdr68, pCS2+GFP, pCS2+mRFP1, pEGFPC2-Wdr68, pCS2+GFPWdr68, pCS2-mRFP1-Dyrk1a and pCS2+Dyrk1b2256 plasmids were previously described [1], [2]. The plasmids pGL3-mgn-luc and pEMSV-MyoD used in the transient transfection reporter assays were previously described [34]. The pCS2+GFPNESWdr68 plasmid was constructed by PCR using primer NES-f1 5′- GCAGAATTCGCCAACCTGGTCGATTTACAGAAGAAGCTAGAAGAATTAGA.

ATTAGACGAACAACAAGGAGGTATGTCGTTGCACGGTAAACG-3′ and CS2+rev 5′- CTATAGTTCTAGAGGCTCGAGCTACACCCGCAGGAT-3′ and subcloned into the pCS2+GFP plasmid. The Cebp fragment was PCR amplified from ZFIN zlone CB1070 using primers Cebp1-f1 5′-ttcttcatcgatccaccATGTCGGTGTCTGACAACATC-3′ and Cepb1-r1 5′-ttcttcgaattcGCGCACAGGCGGAGCGCAGACG-3′ and subcloned into pCS2+FlagWdr68 to yield plasmid pCS2+CebpFlagWdr68. The human Mad fragment was PCR amplified from pSPMadN35GAL [24] using primers hMAD1-f1 5′-ttcttcatcgatCCACCatggcggcggcggttcggatgaacatccag-3′ and hMAD1-r1 5′-ttcttcgaattcggtaacatggaggcataaccatgttcagc-3′ and subcloned into pCS2+FlagWdr68 to yield plasmid pCS2+MadFlagWdr68. The pCS2+mRFP1-Dyrk1b plasmid was constructed by subcloning a EcoRI-XhoI fragment from pCS2+Dyrk1b2256 into the pCS2+mRFP1 plasmid. All constructs were verified by DNA sequencing.

### RNA Rescue Assays, Embryo Injections and Alcian Blue Staining

Capped mRNAs were synthesized using the mMESSAGE mMACHINE kit as described by the manufacturer (Ambion). Animals were injected with various combinations of 50 ng/uL mRNAs and/or 200 uM morpholinos using pulled glass needles and a picospritzer as previously described [1]. The *wdr68*, *dyrk1b* and respective control morpholinos were previously described [1], [2]. The *wdr68* ^hi3812^ mutant line was previously described [1], [35]. Following injections, animals were allowed to develop until 4 days post fertilization and then processed for alcian blue staining as previously described [1]. Animals were visually inspected on a dissection microscope and scored for the severity of the defects in Meckels and Palatoquadrate formation. Data was analyzed and graphed using Excel. One-way ANOVA tests with a significance level of 0.05 were used as indicated in figures.

### Transient Transfections, Subcellular Localization, and Reporter Assays

C2C12 cells were obtained from the ATCC. Cells were incubated at 37°C in a humidified incubator in an atmosphere of 5% CO_2_ in air. Approximately 9×10^3^ C2C12 cells were plated in growth medium (GM) (DMEM, 2% FBS, 10% cosmic calf serum (Thermo Scientific, HyClone), 100 µg/mL Pen:strep (MP Biomedicals)) in 8-well Poly-D-Lysine coated tissue culture slides (BD BioCoat). Transient transfections with various plasmid combinations were performed 24 hours after plating using FuGene6 transfection reagent according to manufacturers suggestions (Roche). Cells were fixed in 200 µL of 4% paraformaldehyde in PBS for 20 min at 37°C, counterstained with mounting medium containing DAPI (Vector Labs) and visualized by confocal microscopy (Olympus Fluoview 5000) approximately 24 hours post-transfection. For differentiation, cells were switched to differentiation medium (DM) (DMEM, 2% horse serum (Thermo Scientific, HyClone), 100 µg/mL Pen:strep (MP Biomedicals)) at 24 hours post-transfection and were allowed to differentiate for 48 hours before processing for imaging.

Zebrafish embryos injected with GFPWdr68 or GFPNESWdr68 mRNA were fixed at 8hpf stage with 4%PFA in PBS for 30 minutes at room temperature, washed once with PBS, dissected from chorions in PBS+0.1% Tween-20, and then incubated on ice for 10 minutes in PBS+0.1% Triton X-100. Embryos were de-yolked and mounted on slides, counterstained with mounting medium containing DAPI (Vector Labs), coverslipped, and imaged by confocal microscopy.

For the transient transfection reporter assays, approximately 2.5×10^4^ C2C12 cells were plated in triplicate in GM in 24-well tissue culture plates. Transient transfection was done immediately after plating the cells using Lipofectamine 2000 reagent (Invitrogen). Transfected cells in GM were harvested at a confluency of 80–90%. For differentiation, transfected cells at a confluency of 80–90% were switched to DM and allowed to differentiate 48 hours before harvesting. All samples were then processed essentially as described by the manufacturer (Dual-Luciferase Reporter Assay kit, Promega) and assayed using a Turner Designs TD-20/20 luminometer. Relative Light Units (RLU) are Firefly luciferase activity divided by Renilla luciferase activity normalized to the basal GM value. Reporter assays were performed independently 3 or more times.

### Protein-protein Interaction Assays

Co-immunoprecipitation assays were performed with mixtures of S35-methionine-labelled proteins, which were made with the TNT Quick-Coupled Transcription/Translation System kit as instructed by the manufacturer (Promega), with modifications as previously described [2].

### RT-PCR

Total RNA was extracted from C2C12 cells by the TRIzol reagent method according to the manufacturer’s protocol (Invitrogen). Purified total RNA was then treated with RNase-free DNase to eliminate genomic DNA contamination (Promega). First strand cDNA was synthesized using the AccuScript High Fidelity 1^st^ strand cDNA synthesis Kit with oligo dT primers according to the manufacturers recommendations (Agilent Stratagene). PCR was then performed to detect *dyrk1b*, *wdr68*, *myog* and *gapdh*. The forward and reverse PCR primers were designed against target gene mRNA spanning at least one intron (except *gapdh*) to avoid amplification from the presence of contaminating genomic DNA. Mouse *wdr68* was amplified using primer mWdr68-F1, 5′-CGAAACACCTTTGACCACCCGTACC-3′ and mWdr68-R1, 5′-GGCGGAGGTCAAACATTCTCACAGA-3′. Mouse *dyrk1b* was amplified using primer mDyrk1b-FW, 5′- ACCTCCGCCATCTGGAACATAGCAC-3′ and mDyrk1b-RV, 5′-ACGTGAGGTGCCACCAACACTACAC-3′. Mouse *myog* was amplified using primer mMyog-FW 5′-GAGCGCGATCTCCGCTACAGAGG-3′ and mMyog-RV 5′-CTGGCTTGTGGCAGCCCAGG-3′. Mouse *gapdh* was amplified using primer mGAPDH-FW 5′-ACCCAGAAGACTGTGGATGG-3′ and mGAPDH-RV 5′-CCCTGTTGCTGTAGCCGTAT-3′.

### Retroviral Transductions for Antisense Knockdowns

The *dyrk1b*-KD and *wdr68-*KD transductions were performed separately using Mission shRNA Lentiviral particles (Sigma-Aldrich). For *dyrk1b* depletion, validated clone #3025 was used. For *wdr68* depletion, validated clones #4646 and #4650 targeting different *wdr68* mRNA sequences were used (Sigma-Aldrich). The non-target (nt) shRNA control transduction particles contain a sequence that targets no known mouse gene and served as the negative control for all samples. 1.6×10^4^ C2C12 cells/well were plated in 96-well tissue culture plates under GM conditions for 24 hours. Cells were transduced with 20 µL of lentiviral particles at a multiplicity of infection (MOI) ∼1 in the presence of hexadimethrine bromide (Santa Cruz biotech) 8 µg/mL for 18 hours. Cells were then transferred to 6-well tissue culture plates and allowed to recover for 24 hours in virus-free GM followed by selection for puromycin resistance at 1.5 µg/mL (Invivogene).

### Western Blot Quantification

Cells were collected 6 days post-viral transduction and were washed twice with room temperature PBS. The C2C12 cells were then homogenized in ice-cold lysis buffer (1% NP40, 0.5% sodium deoxycholate, 0.1% SDS, 1X PBS, with 1X Protease Inhibitor Cocktail, BD Biosciences), and gently rocked for 15 min at 4°C. Adherent cells were then removed with a cell scraper, transferred to a microcentrifuge tube and incubated for 1 hour on ice. Cell lysates were then centrifuged for 10 min at 10,000 *g* at 4°C to remove insoluble debris. Supernatants were quantified with Coommasie Plus Bradford assay reagent (Thermo Scientific). 14–18 µg of cell lysates were ran on SDS-PAGE gels and then transferred onto PVDF membrane (Thermo Scientific). Primary antibodies used were anti-DCAF7/Wdr68 antibody (Sigma-Aldrich), anti-Myogenin 5FD (Santa Cruz Biotechnology), anti-β tubulin G-8 (Santa Cruz Biotechnology) followed by secondary detection with appropriate HRP-conjugated antibodies. Proteins were detected by enhanced chemiluminescence using a VersaDoc 5000 MP (Bio-Rad) and quantified in ImageLab. Data was analyzed and graphed in Excel.

### Immunocytochemistry

Immunocytochemistry was carried out with 8-well CC2 coated cell culture slides (LAB TEK). 2×10^4^ cells of the various C2C12 knockdowns were seeded per well and allowed to differentiate for 48 hours under DM conditions. Cells were then fixed, permeabilized and blocked. Cells were then treated with primary anti-Myog F5D antibody overnight at 4°C followed by FITC-conjugated horse anti-mouse secondary antibody FI-2000 (Vector Labs). Cells were counterstained with mounting medium containing DAPI for detection of cell nuclei (Vector Labs). All specimens were observed and photographed by confocal microscopy (Olympus Fluoview 5000). Myog+ cells were manually counted across more than 3 different experiments. Data was analyzed and graphed in Excel.

## Supporting Information

Figure S1 The GFPNESWdr68 fusion redistributes to the cytoplasm in zebrafish. A, D) DAPI stained cell nuclei. B, E) GFP fusion proteins. C, F) Overlays of blue and green channels. A–C) Moderate nuclear enrichment of GFPWdr68 (GW) in cells of late epiboly stage zebrafish embryos. D–F) Predominant nuclear exclusion of GFPNESWdr68 (GNESW) in cells of late epiboly stage zebrafish embryos.
(TIF)

Figure S2 The MadFlagWdr68 fusion can physically interact with Dyrk1b. Lanes 1–4 show 2% of the proteins included in the co-immunoprecipitation reactions. Lanes 5–9 show the co-immunoprecipitated products. Lane 5, the negative control Luciferase protein does not interact with FlagWdr68. Lane 6, Dyrk1b interacts with FlagWdr68. Lane 7, Dyrk1b does not co-immunoprecipitate in the absence of FlagWdr68. Lane 8, Dyrk1b interacts with MadFlagWdr68. Lane 9, Luciferase does not interact with MadFlagWdr68.
(TIF)

Figure S3 The *wdr68* gene is expressed in C2C12 cells. RT-PCR analysis was performed on C2C12 cells in growth medium (GM) and after 48 hours in differentiation medium (DM). The *dyrk1b*, *wdr68* and *gapdh* genes are expressed in both GM and DM. The *myog* gene is not expressed in GM but is readily detected after 48 hours in DM.
(TIF)

Figure S4 shRNA knockdown of *wdr68* in C2C12 cells. Quantitative western blotting revealed an average 50% reduction of *wdr68* in *wdr68*-KD1 cells and an average 78% reduction of *wdr68* in *wdr68*-KD2 cells relative to the *wdr68* expression level in nt control cells. Expression levels of *wdr68* were normalized to the expression of ß-tubulin within the same lane. Samples shown are from the same gel but from non-adjacent lanes.
(TIF)

Table S1 A nuclear export signal impedes Wdr68 function in craniofacial development. Percent of animals on the left, number of animals on the right, for all trials combined. Presence of intact Meckel’s (M) cartilage and Palatoquadrate (PQ) cartilages served as the basis for scoring. GW = GFPWdr68, GNESW = GFPNESWdr68.
(DOCX)

Table S2 A transcriptional repression domain impairs Wdr68 function in craniofacial development. Percent of animals on the left, number of animals on the right, for all trials combined. Absence of intact Meckel’s (M) cartilage and Palatoquadrate (PQ) cartilages served as the basis for scoring. FW = FlagWdr68, MFW = MadFlagWdr68, CFW = CebpFlagWdr68.
(DOCX)
