# Supplementary figures and images for: Wdr68 Requires Nuclear Access for Craniofacial Development
Source: PLoS One. 2013 Jan 22;8(1):e54363. doi: 10.1371/journal.pone.0054363 (PMC3551808; doi:10.1371/journal.pone.0054363)

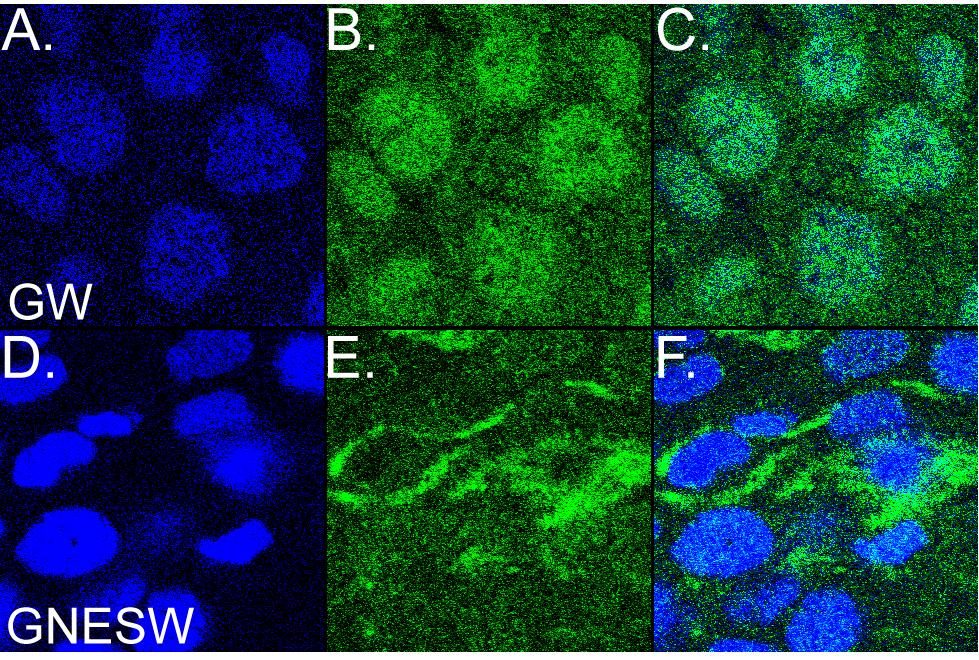

Supplement: Figure S1 — The GFPNESWdr68 fusion redistributes to the cytoplasm in zebrafish. A, D) DAPI stained cell nuclei. B, E) GFP fusion proteins. C, F) Overlays of blue and green channels. A–C) Moderate nuclear enrichment of GFPWdr68 (GW) in cells of late epiboly stage zebrafish embryos. D–F) Predominant nuclear exclusion of GFPNESWdr68 (GNESW) in cells of late epiboly stage zebrafish embryos. (TIF) [file pone.0054363.s001.tif]

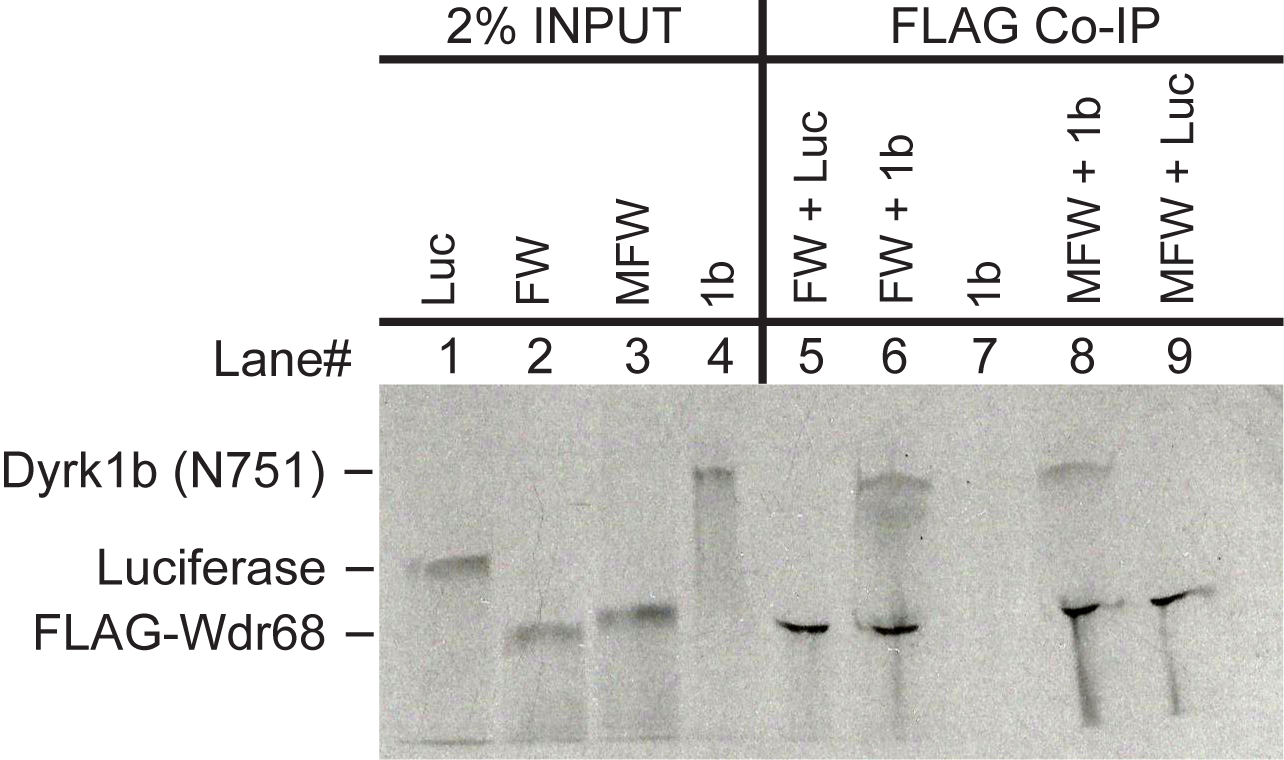

Supplement: Figure S2 — The MadFlagWdr68 fusion can physically interact with Dyrk1b. Lanes 1–4 show 2% of the proteins included in the co-immunoprecipitation reactions. Lanes 5–9 show the co-immunoprecipitated products. Lane 5, the negative control Luciferase protein does not interact with FlagWdr68. Lane 6, Dyrk1b interacts with FlagWdr68. Lane 7, Dyrk1b does not co-immunoprecipitate in the absence of FlagWdr68. Lane 8, Dyrk1b interacts with MadFlagWdr68. Lane 9, Luciferase does not interact with MadFlagWdr68. (TIF) [file pone.0054363.s002.tif]

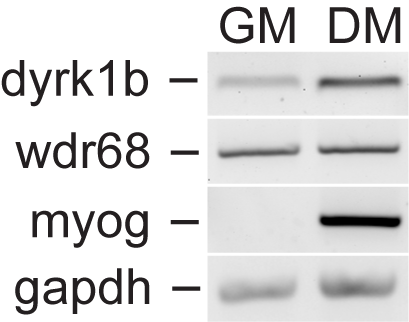

Supplement: Figure S3 — The wdr68 gene is expressed in C2C12 cells. RT-PCR analysis was performed on C2C12 cells in growth medium (GM) and after 48 hours in differentiation medium (DM). The dyrk1b, wdr68 and gapdh genes are expressed in both GM and DM. The myog gene is not expressed in GM but is readily detected after 48 hours in DM. (TIF) [file pone.0054363.s003.tif]

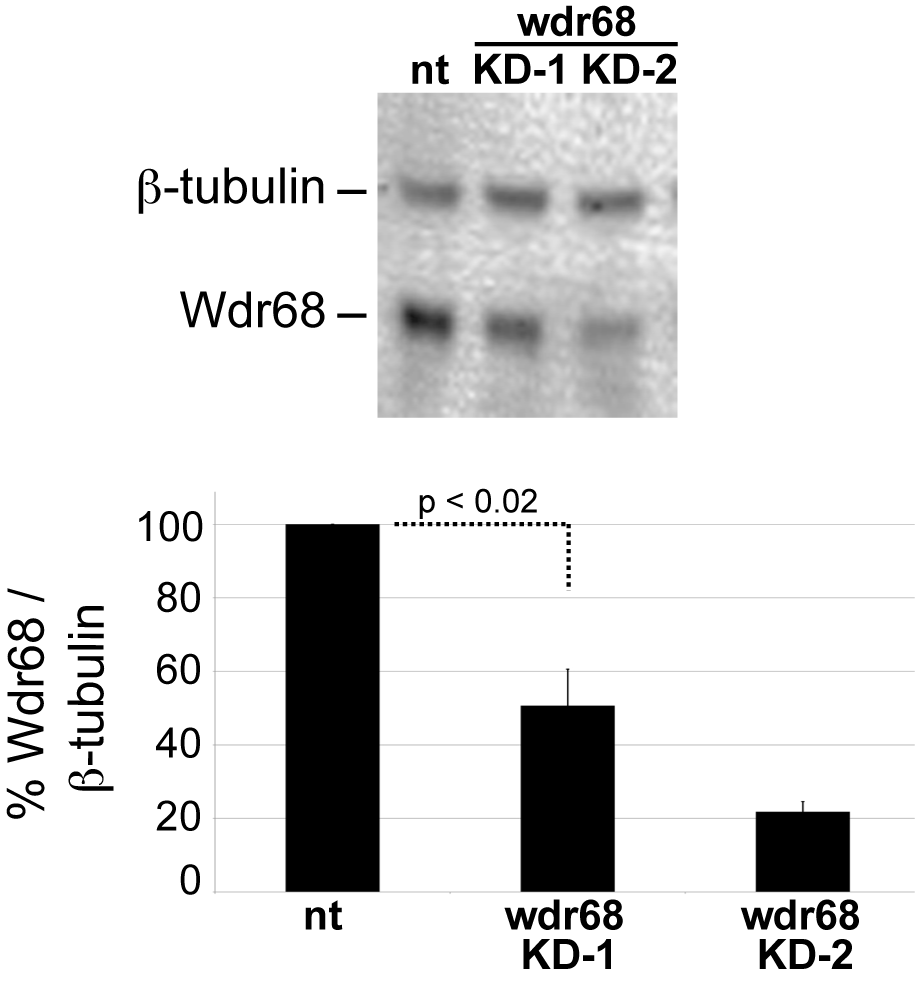

Supplement: Figure S4 — shRNA knockdown of wdr68 in C2C12 cells. Quantitative western blotting revealed an average 50% reduction of wdr68 in wdr68-KD1 cells and an average 78% reduction of wdr68 in wdr68-KD2 cells relative to the wdr68 expression level in nt control cells. Expression levels of wdr68 were normalized to the expression of ß-tubulin within the same lane. Samples shown are from the same gel but from non-adjacent lanes. (TIF) [file pone.0054363.s004.tif]
