# Supplementary material for: Wdr68 Requires Nuclear Access for Craniofacial Development
Source: PLoS One. 2013 Jan 22;8(1):e54363. doi: 10.1371/journal.pone.0054363 (PMC3551808; doi:10.1371/journal.pone.0054363)
Supplement: Table S1 — A nuclear export signal impedes Wdr68 function in craniofacial development. Percent of animals on the left, number of animals on the right, for all trials combined. Presence of intact Meckel’s (M) cartilage and Palatoquadrate (PQ) cartilages served as the basis for scoring. GW = GFPWdr68, GNESW = GFPNESWdr68. (DOCX) [file pone.0054363.s005.docx]

**Table S1. A nuclear export signal impedes Wdr68 function in craniofacial development.**

| injection condition | M/PQ present | |
| --- | --- | --- |
| ctrl | 100% | 50/50 |
| MO+GFP | 18% | 47/257 |
| MO+GW | 61% | 212/350 |
| MO+GNESW | 18% | 71/385 |
